# Improvement of eosinophilic chronic rhinosinusitis after infection with severe acute respiratory syndrome corona virus 2 during dupilumab therapy: A case report

**DOI:** 10.3389/falgy.2023.1053777

**Published:** 2023-02-02

**Authors:** Mariko Koike-Ieki, Ryoji Kagoya, Makiko Toma-Hirano, Yuko Sasajima, Ken Ito

**Affiliations:** ^1^ Department of Otolaryngology, Faculty of Medicine, Teikyo University, Tokyo, Japan; ^2^ Department of Otorhinolaryngology-Head and Neck Surgery, Graduate School of Medicine, The University of Tokyo, Tokyo, Japan; ^3^ Department of Pathology, Teikyo University School of Medicine, Tokyo, Japan

**Keywords:** eosinophilic chronic rhinosinusitis, dupilumab, severe acute respiratory syndrome corona virus 2, cytokine balance, dysosmia

## Abstract

Eosinophilic chronic rhinosinusitis (ECRS) is an intractable type 2 inflammatory disease of the paranasal sinuses that persists even after endoscopic sinus surgery (ESS) and systemic corticosteroid therapy. Dupilumab, a monoclonal antibody against the shared receptor components of interleukin (IL)-4 and IL-13, is a novel and effective treatment option for ECRS. Herein, an atypical case of ECRS that improved after infection with severe acute respiratory syndrome corona virus 2 (SARS-CoV-2) during dupilumab therapy is reported. A 40-year-old man with a history of ESS for ECRS visited our hospital with complaints of nasal congestion and dysosmia. Nasal endoscopy revealed bilateral nasal polyps occupying the nasal cavity. Computed tomography (CT) revealed a soft tissue density lesion filling all sinuses on both sides. Based on these findings, ECRS recurrence was confirmed; however, 3 years of subsequent corticosteroid therapy did not improve disease activity. Accordingly, dupilumab therapy was initiated, although 6 months of therapy resulted in only slight improvement in ECRS. Eight months after the initiation of dupilumab therapy, the patient was infected with SARS-CoV-2; thereafter, he noticed an improvement in smell. Nasal endoscopy and sinus CT revealed a marked reduction in nasal polyps and soft tissue density lesions of the sinuses, respectively. With continued dupilumab therapy, no re-exacerbation of ECRS was confirmed at the 6-month follow-up from SARS-CoV-2 infection. Currently, there are no reports describing the impact of SARS-CoV-2 infection on ECRS. As such, careful follow-up and accumulation of cases are necessary.

## Introduction

Chronic rhinosinusitis (CRS) is a multifactorial disorder characterized by chronic inflammation of the paranasal sinuses, in which various symptoms, including dysosmia, nasal obstruction, and mucinous rhinorrhea persist for >12 weeks (1). CRS is phenotypically classified as CRS with nasal polyps (CRSwNP) or CRS without nasal polyps (CRSsNP) based on nasal endoscopic findings (1). In Europe and the United States, CRSwNP is characterized by eosinophilic infiltration of nasal polyps (1). In East Asian countries, CRSwNP is classified as eosinophilic CRS (ECRS) and non-ECRS, depending on the severity of eosinophilia in the nasal tissue, because the extent of eosinophil infiltration is variable (2). Patients are clinically diagnosed with ECRS by Japanese Epidemiological Survey of Refractory Eosinophilic Chronic Rhinosinusitis (JESREC) score ≥11 (2). Histological diagnosis is made by nasal tissue eosinophil count ≥70/high-power field (hpf) (2). ECRS is a type 2 inflammatory disease, with infiltration of eosinophils and basophils (3), M2 macrophages (4), and group 2 innate lymphoid cells (5) in the nasal tissue, accompanied by inflammation of the olfactory mucosa and increased levels of type 2 cytokines, including interleukin (IL)-4, IL-5, and IL-13 (6). Dupilumab is a fully human VelocImmune-derived (Regeneron Pharmaceuticals Inc., Westchester County, NY, USA) monoclonal antibody that blocks the shared receptor components of IL-4 and IL-13, which are key and central drivers of type 2 inflammation (7). Dupilumab can reduce nasal polyps and improve nasal symptoms, and has been approved in Japan as an add-on maintenance treatment in adult patients with intractable ECRS (8).

Since March 2020, outbreak of coronavirus disease 2019 (COVID-19) has caused various changes in the daily practice of otorhinolaryngology. Severe respiratory viral infections are presumed to affect ECRS through changes in systemic cytokine levels (9). However, there are no reports describing the impact of severe acute respiratory syndrome coronavirus 2 (SARS-CoV-2) infection on ECRS. Herein, we report a case of ECRS that improved after SARS-CoV-2 infection during dupilumab therapy.

## Case presentation

**Figure 1:**
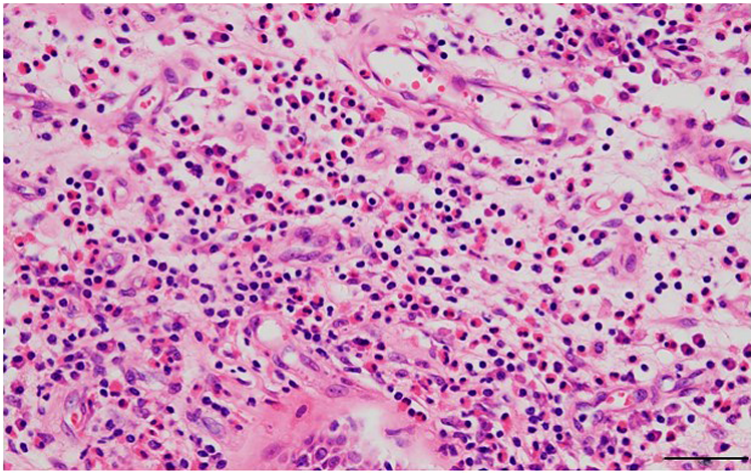
Hematoxylin and eosin-stained section of the nasal polyp (scale bar = 50 *μ*m). Marked eosinophil infiltration in the subepithelial area is observed.

**Figure 2:**
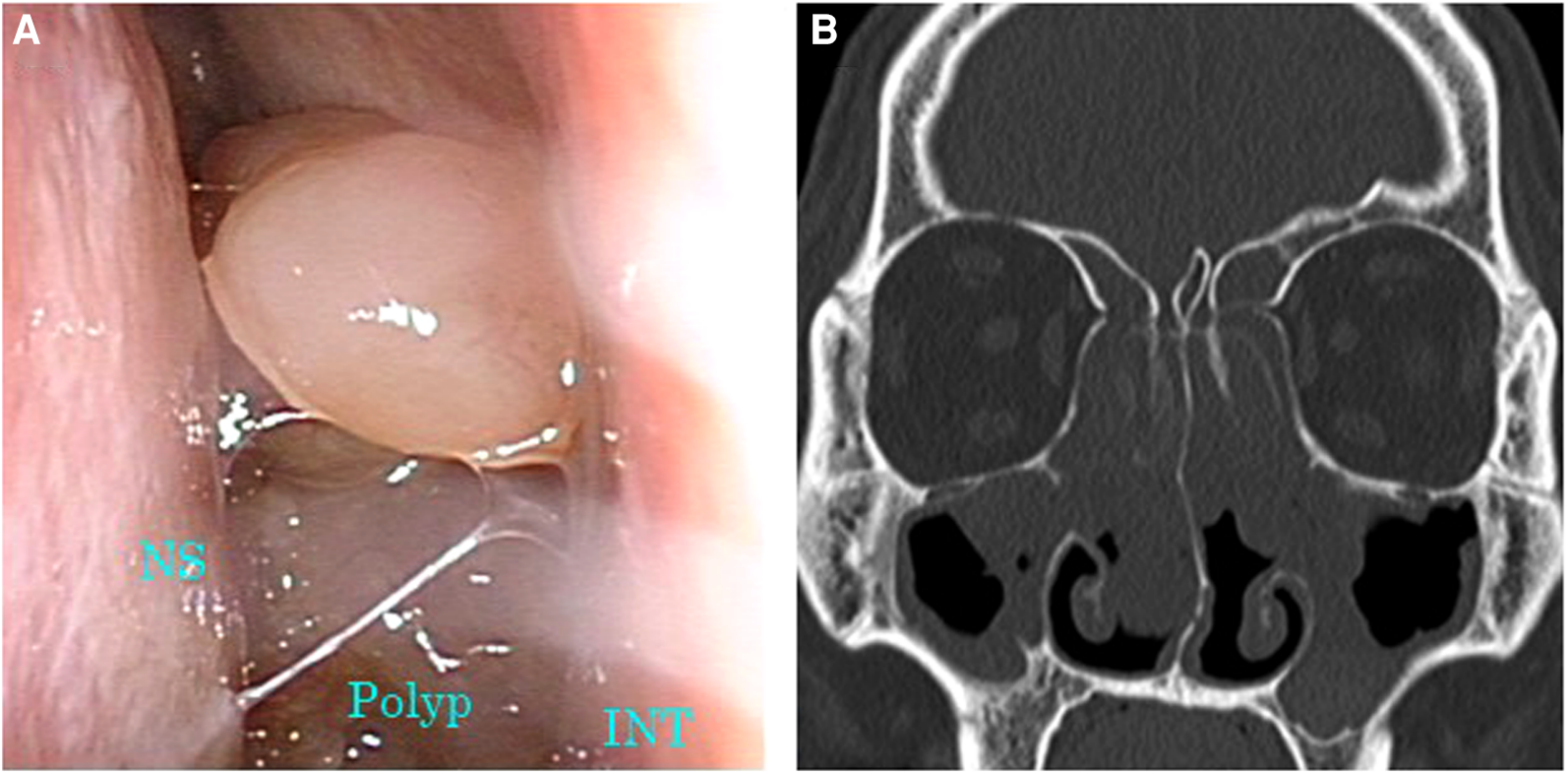
Images of nasal endoscopy and sinus CT before initiation of dupilumab therapy. (**A**) Nasal polyps occupying the common nasal meatus were observed. (**B**) Soft tissue density lesions were filling all sinuses on both sides. CT, computed tomography; INT, inferior nasal turbinate; NS, nasal septum.

A 40-year-old man with ECRS, who had undergone endoscopic sinus surgery (ESS) in our hospital 8 years previously and had been confirmed to have a recurrence of ECRS 2 years after the surgery, visited the same department for the first time in a long time with complaints of worsening nasal congestion and dysosmia. JESREC score and tissue eosinophil count in the nasal polyp at the first treatment was 17 and 107/hpf, respectively (Figure 1). On nasal endoscopy, nasal polyps occupying both sides of the common nasal meatus were observed, with a total polyp score (TPS) of 8 (Figure 2A) (10). Computed tomography (CT) of the sinus revealed a soft tissue density lesion filling all sinuses on both sides, with a Lund-Mackay CT score (LMS) of 24 (Figure 2B) (11). The blood eosinophil percentage was 8%. The self-administered odor questionnaire score (12) was 0/40 (0%). The patient also had non-steroidal anti-inflammatory drug-exacerbated respiratory disease, and was treated with a combination of a long-acting beta-agonist and inhaled corticosteroids in the Department of Respiratory Medicine of our hospital for the past 10 years. There had been no asthma attacks or deterioration of respiratory function for several years. Because an exacerbation of recurrent ECRS was confirmed, multiple treatments with a combination of systemic and intranasal corticosteroids for one month were administered intermittently for 3 years. However, the symptoms only slightly improved during treatment.

**Figure 3:**
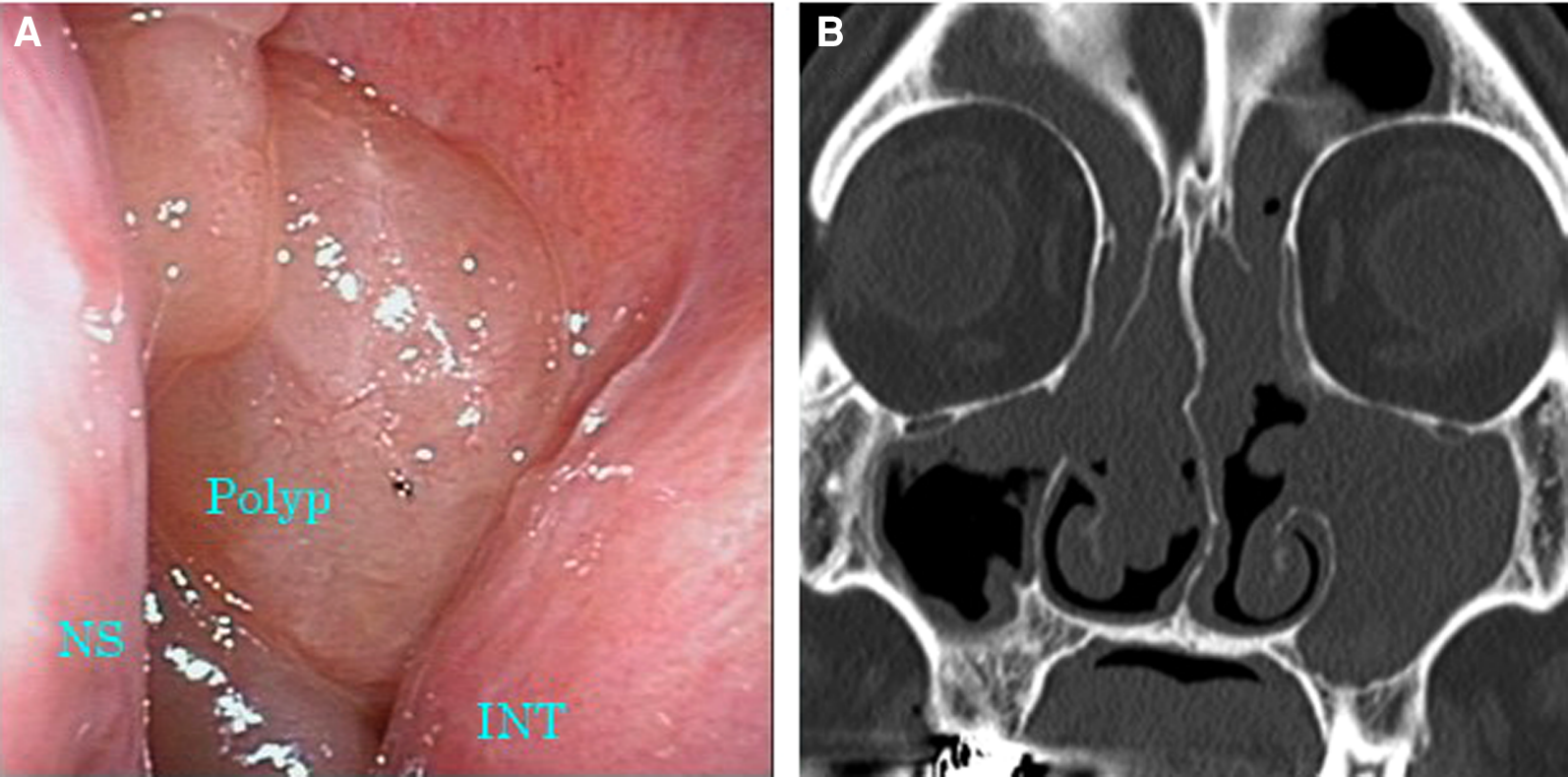
Images of nasal endoscopy and sinus CT 6 months after the initiation of dupilumab therapy. (**A**) Nasal polyps occupying the common nasal meatus were still observed. (**B**) Soft tissue density filled all sinuses on both sides. CT, computed tomography; INT, inferior nasal turbinate; NS, nasal septum.

**Figure 4:**
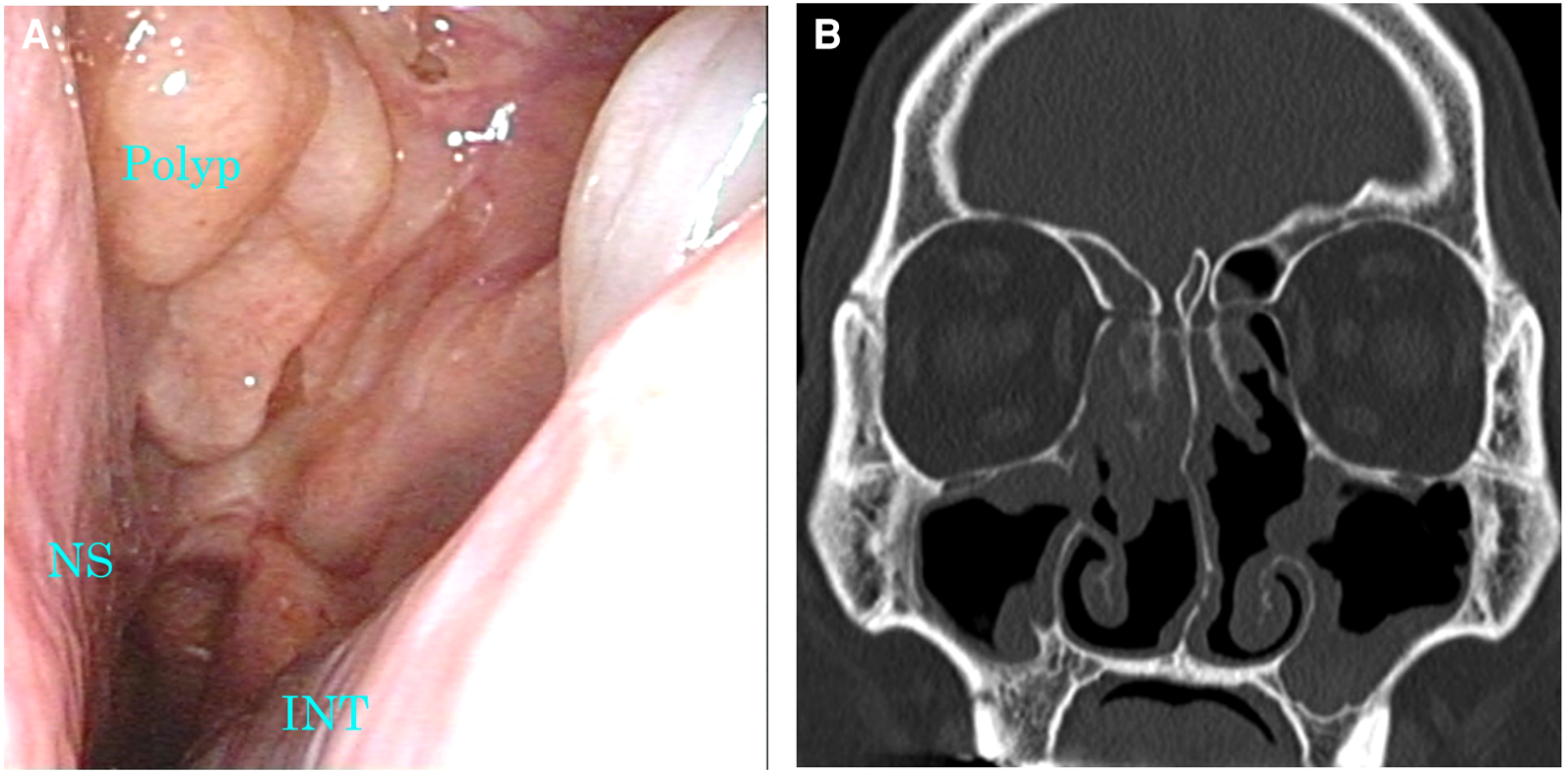
Images of nasal endoscopy and sinus CT 1 month after the diagnosis of SARS-CoV-2 infection. (**A**) A marked reduction of nasal polyps was confirmed. (**B**) Soft tissue density lesions in the sinuses were markedly reduced. CT, computed tomography; INT, inferior nasal turbinate; NS, nasal septum; SARS-CoV-2, severe acute respiratory syndrome coronavirus 2.

Considering the continuous nasal congestion and dysosmia due to nasal polyposis, despite the history of ESS and repeated courses of systemic and intranasal corticosteroids, the patient was indicated to receive dupilumab. Subcutaneous injection of dupilumab 300 mg every 2 weeks for 6 months resulted in slight improvement in nasal polyposis without the use of corticosteroids, with an LMS of 22 and a TPS of 6 (Figures 3A,B). The blood eosinophil percentage at that time was 11%. Eight months after initiation of dupilumab therapy, the patient developed a sore throat and slight fever and tested positive for SARS-CoV-2 infection based on a polymerase chain reaction test on the 3rd day of onset. He took oral loxoprofen and tranexamic acid for 7 days and the symptoms disappeared. No targeted drug therapy for SARS-CoV-2 or systemic steroids were needed. One month after SARS-CoV-2 infection, the patient noticed that his sense of smell had improved compared to that before the infection. A marked reduction in nasal polyps was confirmed on nasal endoscopy, with a TPS of 3 (Figure 4A). Sinus CT also revealed a marked reduction in soft tissue density lesions of the paranasal sinuses, with an LMS of 12 (Figure 4B). The blood eosinophil percentage 1 year from the initiation of the dupilumab therapy was 7%. With continued dupilumab therapy, no re-exacerbation of ECRS was confirmed at the 6-month follow-up from the infection.

## Discussion

ECRS is a type 2 inflammatory disease of the paranasal sinuses, some of which are intractable. Although ESS is an effective treatment for ECRS, approximately 60% of patients experience recurrence after surgery and often require multiple interventional procedures (13). Systemic corticosteroid therapy is also effective in treating ECRS. However, long-term administration is not desirable because side effects, such as diabetes, osteoporosis, and increased intraocular pressure, can occur (14).

Dupilumab is a biological drug that blocks the shared receptor component of IL-4 and IL-13 (7) and has been reported to be effective for type 2 inflammatory diseases including atopic dermatitis, bronchial asthma, and ECRS (15). A previous study reported that patients with ECRS treated with dupilumab experienced clinically meaningful improvements in dysosmia within 2 weeks of the start of therapy (15). Another study reported that patients with ECRS exhibited significant improvement in various endpoints, including nasal polyp score and total symptom score within 8 weeks from the first administration of dupilumab (16). Based on these reports, the effects of dupilumab appear at a relatively early stage.

In the present case, the size of the nasal polyps and sinus CT findings improved only slightly, and dysosmia remained unchanged for 8 months after the initiation of dupilumab therapy. At 9 months from the first administration, the patient was infected with SARS-CoV-2 and, after the infection, both objective findings and subjective dysosmia improved dramatically. Although the exact mechanism of improvement is unclear, it may be attributable to changes in cytokine balance. In one report, type 1 cytokines including IL-6, IL-1β, and interferon-gamma (IFN-*γ*) levels were systemically elevated in SARS-CoV-2 infected patients (9). IFN-*γ* is also known to attenuate respiratory eosinophilic inflammation (17). These findings suggest that SARS-CoV-2 infection may have contributed to the relief of ECRS. Licata et al. (18) reported 2 cases of atopic dermatitis that improved after asymptomatic or slightly symptomatic SARS-CoV-2 infection. They also discussed the possibility that an increased type-1 immune response with a simultaneous decrease in type-2 immune response may contribute to changes in the state of atopic dermatitis.

To our knowledge, no studies have addressed the impact of SARS-CoV-2 infection on ECRS. On the other hand, ECRS is reported to protect against SARS-CoV-2 infection and the following mechanism is proposed: type-2 cytokines downregulate angiotensin converting enzyme 2, an entry receptor for SARS-CoV-2 (19).

There is a limitation that should be recognized in this report. We cannot rule out the possibility that dupilumab showed delayed effect at 9 months from the initiation of therapy, albeit previous studies (15, 16) reported the effect of dupilumab is generally confirmed within 8 weeks from the initiation.

We should also note that the improvement triggered by SARS-CoV-2 infection was maintained for at least 6 months thereafter despite the transient infection. Sustained remission of ECRS after SARS-CoV-2 infection may be due to delayed effect of dupilumab therapy. On the other hand, an animal study reported that immunological changes caused by SARS-CoV-2 infection persist for some time, even if the infection disappeared (20). In the present case, changes in cytokine balance caused by infection may have persisted.

## Conclusion

We report a case of ECRS that improved after the patient acquired SARS-CoV-2 infection during dupilumab therapy, with subsequent maintenance of the mild condition of the disease for 6 months. This report adds to the current knowledge base by suggesting that severe respiratory tract infections may affect the efficacy of dupilumab in ECRS. The long-term course of dupilumab-introduced ECRS cases is not well known; therefore, careful observation and accumulation of a greater number of cases are necessary.

## Data Availability

The original contributions presented in the study are included in the article/Supplementary Material, further inquiries can be directed to the corresponding author/s.
